# Morphological change in cranial shape following the transition to agriculture across western Eurasia

**DOI:** 10.1038/srep33316

**Published:** 2016-09-13

**Authors:** Olivia Cheronet, John A. Finarelli, Ron Pinhasi

**Affiliations:** 1 School of Archaeology, University College Dublin, Belfield, Dublin 4, Ireland; 2 Earth Institute, University College Dublin, Belfield, Dublin 4, Ireland; 3 School of Biology and Environmental Sciences, University College Dublin, Belfield, Dublin 4, Ireland

## Abstract

The Neolithic transition brought about fundamental social, dietary and behavioural changes in human populations, which, in turn, impacted skeletal morphology. Crania are shaped through diverse genetic, ontogenetic and environmental factors, reflecting various elements of an individual’s life. To determine the transition’s effect on cranial morphology, we investigated its potential impact on the face and vault, two elements potentially responding to different influences. Three datasets from geographically distant regions (Ukraine, Iberia, and the Levant plus Anatolia) were analysed. Craniometric measurements were used to compare the morphology of pre-transition populations with that of agricultural populations. The Neolithic transition corresponds to a statistically significant increase only in cranial breadth of the Ukrainian vaults, while facial morphology shows no consistent transformations, despite expected changes related to the modification of masticatory behaviour. The broadening of Ukrainian vaults may be attributable to dietary and/or social changes. However, the lack of change observed in the other geographical regions and the lack of consistent change in facial morphology are surprising. Although the transition from foraging to farming is a process that took place repeatedly across the globe, different characteristics of transitions seem responsible for idiosyncratic responses in cranial morphology.

The Neolithic transition marked the shift in human populations from a subsistence spectrum based solely on hunting, fishing and the gathering of natural resources, to strategies involving some dependence on domesticated plants and animals. This marked a major turning point in human prehistory. Resources in a hunting/gathering regime fluctuate temporally and spatially in their abundance, availability and reliability, and although domesticated resources also fluctuate in their availability, their exploitation provided a means for human groups to gradually develop a range of predictable subsistence resources. This process has, in turn, resulted in modifications of many aspects of the human lifestyle, including altered demographic patterns and mating structures^1^, as well as sedentism and modifications in health profiles^2^.

As with all organisms, humans adapt to their environmental conditions, and patterns of associated morphological variation have been identified in the Neolithic transition. This comprises a general, organism-wide trend toward gracilisation, including reduction in stature^3^, long bone composition^4^, and cranial morphology. This last aspect has occasionally been linked to changes in diet, from a tougher hunter-gatherer diet, to a softer farmer diet, with corresponding gracilisation of the masticatory apparatus, notably the mandible and maxilla, as well as areas of muscle attachment^5^,^6^,^7^,^8^.

Changes in masticatory regimes, however, cannot completely account for observed cranial variation among Holocene human populations. Carlson and van Gerven^9^ measured the cranial and mandibular morphology of Nubians across the transition from hunter-gatherer populations to agriculturalists. In addition to changes associated directly with mastication, they also found a decrease in cranial length and increase in cranial height. Similar patterns have been observed to varying degrees in numerous studies using both standard craniometric measurements^5^,^10^,^11^ and geometric morphometrics^6^,^7^. While more research is required in order to assess whether the onset of these morphological changes are directly triggered by a transition to an agricultural life, it is clear that these patterns are lacking in pre-agricultural societies.

Although the complex developmental and functional processes that shape observed morphological variation in the human cranium are not fully understood, an increasing number of elements are being clarified. It is now widely accepted that overall cranial variation follows the expectations of a neutral evolutionary process^12^,^13^,^14^,^15^, and as such, quantified cranial morphology can be employed in a similar manner to genetic data to elucidate questions of population structure^16^,^17^,^18^. Yet specific elements of the skull indicate that non-neutral processes are at play, and adaptive changes can be observed as significant determinants of morphology in the masticatory and facial regions, in response to climate^19^,^20^,^21^ and subsistence^7^,^8^,^22^. Neutral genetic elements appears to dictate a higher proportion of the temporal bone and vault morphology^23^,^24^. As such, investigating detailed morphological variation can allow a deeper understanding of the mechanisms involved, and it is expected that modules within a single cranium may reflect different processes.

Although changes in many elements of lifestyle are associated with the transition to an agriculture-based subsistence, heterogeneity remains present. In a western Eurasian context only, this diversity in the mode of transition is readily observed. Indeed, in some regions, this transition is abrupt with sedentary farmers migrating in from other regions and replacing local hunter-gatherer populations, for example the Linearbandkeramik (LBK) westbound spread^25^. In other instances, the transition was a prolonged process, with local hunter-gatherers adopting selected elements of the agricultural lifestyle gradually, for example, the Pontic region^26^. Here, we investigate cranial morphological changes directly linked to the transition to an agricultural lifestyle in Eurasia. Three regions are specifically considered here: The Levant/Anatolia, Iberia, and the Ukraine. In each, the transition took place in a very different way.

The Levant is the first and primary western Eurasian region in which agricultural communities emerged. In this region, the development of the Neolithic subsistence pattern and lifestyle took place gradually. The first step of this process can be seen in the Natufian culture beginning ~15,000 cal BP. Despite subsisting on wild resources, they engaged in intensive cultivation and processing of wild plants, and were sedentary^27^. This was followed by the Pre Pottery Neolithic cultures (~11,700 to 8,400 cal BP), which show the first evidence of crop domestication (cereals, legumes), followed by the domestication of goat, sheep, cattle and pigs. The reliance on domesticated resources was, however, a gradual process, with a continuation of some reliance on the gathering and cultivation of wild plants, and the hunting of large and small game^28^. It is only in the subsequent Pottery Neolithic cultures that the full agricultural package (domesticated crops, livestock, pottery) can be found^29^. Direct evidence of dietary change following the transition in this region has only been obtained through the study of dental microwear, although no consistent pre/post-transition patterns have been detected^30^,^31^.

In Iberia, the transition from foraging to farming lifestyles is viewed as a complex maritime colonisation process in which Cardial culture farmers establish enclave settlements along various coastal regions^32^. This model, which remains the dominant one, views the transition in Iberia as a cultural replacement by the colonisation of exogenous farmers bringing with them domesticates (plant and animal), pottery technology, and other elements of the Neolithic package by ~7,500 cal BP^32^. Furthermore, a number of stable isotope studies suggest that a marked reduction in the consumption of marine products associated with the transition to an agricultural lifestyle^33^,^34^,^35^,^36^,^37^, although a significant level of variability in carbon and nitrogen isotopic ranges was noted^38^,^39^.

Finally, in the Ukraine, the Mesolithic-Neolithic transition had its roots in the Mesolithic of this area (as indicated by lithic technology and burial rites^26^). Beginning around 7,000 cal BP with the early Dnieper-Donets culture, these populations had a subsistence spectrum that mainly relied on wild resources and were not fully sedentary. Their categorisation as ‘Neolithic’ was mainly based on evidence of the use of pottery, a technology that was most likely obtained through contact with neighbouring fully agricultural cultures, namely the Cucuteni-Triploye^26^. Over the next millennia, by the mid 5^th^ millennium BP, the management of the horse taken together with evidence of cattle and goat domestication, indicates a shift in subsistence to pastoral-farming economy^26^,^40^. Despite these fundamental cultural changes, it is interesting to note that isotopic evidence suggests very little consistent change dietary composition^41^,^42^, with evidence for the consumption of both freshwater and terrestrial resources through prehistory.

This study specifically aims to identify whether the Neolithic transition contains a universal morphological signal, which is reflected in transitions that occurred independently of one another, in regions that are geographically remote with different cultural contexts and with no apparent consistent contrast in pre and post-transitional diets. We test changes in a number of cranial dimensions of populations prior and subsequent to the transition event in three independent geographic regions. While we cannot be certain that there was no cultural contact, and therefore no potential gene flow, between these Eurasian regions, the large distances between the regions and lack of archaeological evidence to support such contacts makes this scenario implausible. We partitioned the skull into facial and vault modules, attempting to isolate the exact nature of the potentially observed change through principal component analyses. From this we demonstrated that, although no consistent structure in facial variability was observed in the PCAs, vault variability is similar in all regions. Furthermore, only one region (Ukraine) exhibited variation directly linked to the change in lifestyle.

## Results

### Description of the morphospace produced by a PCA of pre-transition specimens

#### Vaults

Table 1 summarises the vault variable loadings for the first two principal components of PCAs of the pre-transitional individuals from each location. The first principal component of the three regions reflects a consistence in patterns of morphological variability. Accounting for 54%, 41% and 41% of the Ukrainian, Iberian and Levant morphological variances, respectively, in all cases, both length measurements considered (M1 and M5) load heavily on this component. Furthermore, all breadth variables display an inverse proportionality with the length variables. In both Ukrainian and Iberian vaults the breadth element is mostly represented by variables M8 and M11 (maximum cranial breadth and biauricular breadth, respectively). In the Levant, although loadings for breadth variables are much lower than those of length variables, all vary within the same range (around 0.2), with the exception of M12 (biasterionic breadth). All in all, this suggests that, as PC1 values increase, vaults become shorter and broader in the first two regions, and vice versa in the last. PC2 accounts for 17%, 21% and 22% of the variance in the Ukraine, Iberia and the Levant, respectively, and for each of the regions, variable M17 (basi-bregmatic height) is a significant contributors to the axis, suggesting a decrease in cranial height with increasing values along the axis. Furthermore, this is complemented by variable M9 (least frontal breadth) for the Ukraine and Levant to a lesser extent, reflecting a reduction in this dimension. In Iberia, PC2 is significantly defined by M1 (maximum cranial length). However, M5 (base length), although also significant, affects the axis in the opposite direction, suggesting a proportional elongation of the superior part of the vault.

#### Faces

In contrast to the vault PCAs, the facial data result in much more heterogeneous morphospaces (table 2). The first two components produced by the Ukrainian dataset account for 49% and 36% of the total variance respectively. PC1 is primarily defined by bizygomatic breadth (M45), with all other variables displaying much smaller loadings on this axis. PC2 is principally defined by variation in variable M40 (basion-prosthion length). The same analysis of the Iberian data results in the first two axes accounting for 49% and 26% of the variance, respectively. The former is principally driven by bizygomatic breadth (M45), as in the Ukrainian case, with PC2 indicating a reduction in upper facial height (M48) and nasal height (M55), and an increase in basion-prosthion length. Finally, the Levant dataset is represented by a first principal component (57% of variance) predominantly suggesting a reduction in breadth and increase in height of both orbital and nasal cavities (illustrated by the loadings of variables M51, M52, M54 and M55) and PC2 (25% of variance) by the proportional sizes of these two facial elements. Of the three facial morphospaces described, those obtained for the Ukraine and Iberia share some characteristics, although Levant faces define different variations.

### Distribution of groups within morphospace

**Figure 1:**
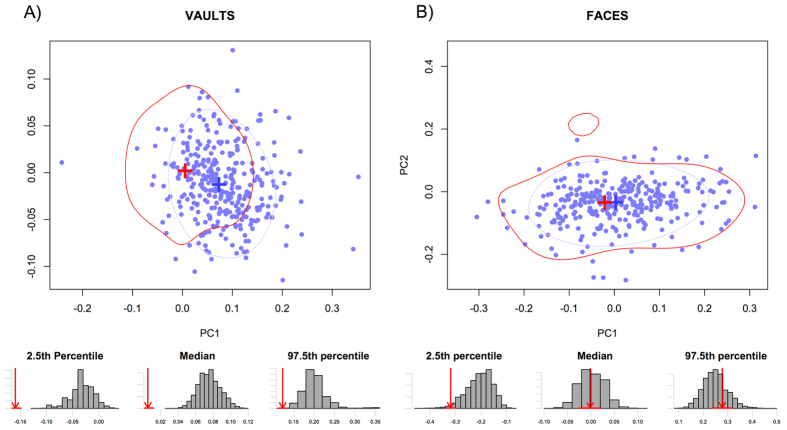
Bivariate plots of the first two principal components projection of the Ukrainian post-transition group on a PCA of the Ukrainian pre-transition group (**A**) Vaults, (**B**) Faces. Blue: distribution of the post-transition. Red: distribution of the pre-transition group. For both groups, the crosses represent the mean shape and contours the distribution of 75% of the data. The histograms below illustrate the differences in pre and post transition groups distributions. For the 2.5 percentile, the mean and the 97.5 percentile, the bars represent the distribution of the larger post-transition resampled to the size of the pre-transition group, and the red arrow the value of the pre-transition group (for which the error associated with the data estimation process is expressed by the red horizontal line).

**Figure 2:**
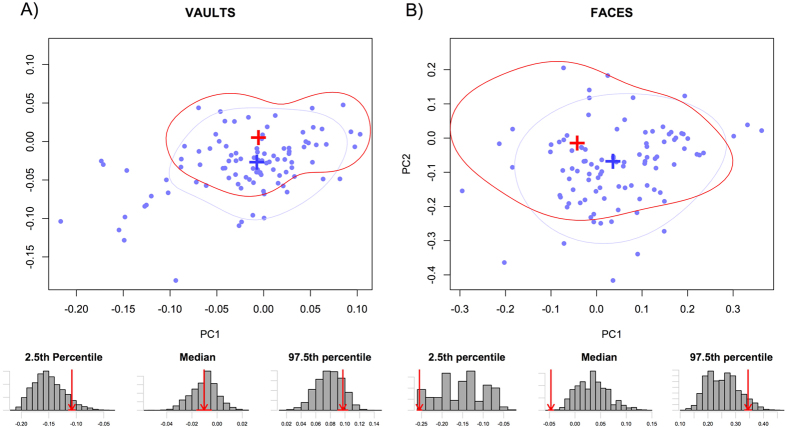
Bivariate plots of the first two principal components projection of the Levant post-transition group on a PCA of the Levant pre-transition group (**A**) Vaults, (**B**) Faces. Blue: distribution of the post-transition. Red: distribution of the pre-transition group. For both groups, the crosses represent the mean shape and contours the distribution of 75% of the data. The histograms below illustrate the differences in pre and post transition groups distributions. For the 2.5 percentile, the mean and the 97.5 percentile, the bars represent the distribution of the larger post-transition resampled to the size of the pre-transition group, and the red arrow the value of the pre-transition group (for which the error associated with the data estimation process is expressed by the red horizontal line).

**Figure 3:**
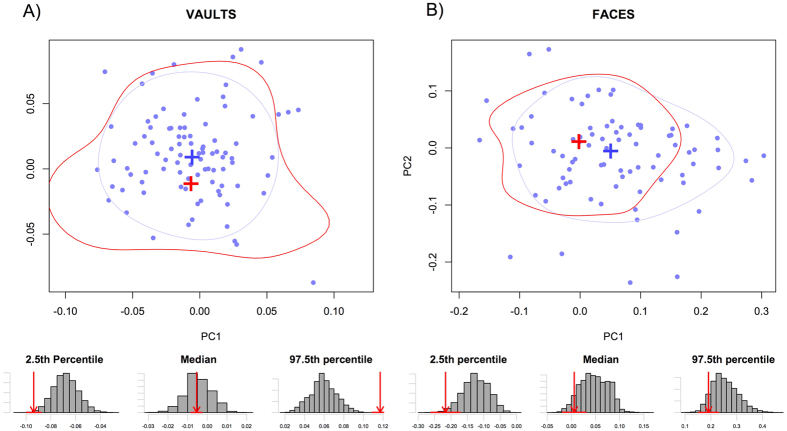
Bivariate plots of the first two principal components projection of the Iberian post-transition group on a PCA of the Iberian pre-transition group (**A**) Vaults, (**B**) Faces. Blue: distribution of the post-transition. Red: distribution of the pre-transition group. For both groups, the crosses represent the mean shape and contours the distribution of 75% of the data. The histograms below illustrate the differences in pre and post transition groups distributions. For the 2.5 percentile, the mean and the 97.5 percentile, the bars represent the distribution of the larger post-transition resampled to the size of the pre-transition group, and the red arrow the value of the pre-transition group (for which the error associated with the data estimation process is expressed by the red horizontal line).

Comparing the distributions of pre and post-transition groups on the morphospaces (Figs 1, 2, 3), it is clear that, for the most part, very little morphological differentiation exists between pre- and post-transition groups, with the exception of the Ukrainian vaults. For the Iberian and Levant samples, the overlap in distributions is most pronounced in PC1. In the case of the Ukrainian vaults (Fig. 1) however, strong evidence points towards a shift of distribution towards higher PC1 values upon the transition. This pattern appears to exist in both males and females (Supplementary Figures S7 and S8), and can clearly be seen in the distributions of resampled data. Whereas Ukrainian vaults show no overlap in any of the measures shown (2.5^th^ and 97.5^th^ percentiles, and median), all other datasets have overlapping distributions.

## Discussion

There is little consistence in patterns of cranial morphological change linked to the transition to an agricultural lifestyle in the three western Eurasian populations examined, in either the morphology of the face or vault. Furthermore, we observe a complete lack of patterning in faces, as even the morphospaces described by the three regions were different. Conversely, we detect a significant shift between pre and post-agriculturalists in Ukrainian vaults.

Lack of consistency in facial morphological variation corroborates several previous studies^9^,^11^,^22^,^43^. Although some studies have detected differences between the mandibles of hunter-gatherers and agriculturalists in both modern^7^ and archaeological contexts^44^, the effect of this transition on facial morphology is less clear. During the masticatory process, musculature linking the mandible to the lateral parts of the face (e.g., the zygomatic region) is activated, and strains resulting from this process are felt throughout the facial skeleton^45^,^46^. Nevertheless, while a number of studies have found significant differences in facial robusticity potentially associated with masticatory functions^43^ between hunter-gatherers and agriculturalists in Nubia^9^, the Levant^47^, and Europe and North Africa^22^, others have failed to recognise such patterns at both regional^11^ and global scales^43^ in overall facial morphology. Consequently, it is clear that the response to this type of subsistence change is a process highly localised within the cranial structure, influenced by a multitude of environmental and genetic variables, as illustrated by the findings of González-José *et al*.^11^ of a differentiation of the masticatory region only.

Our results do suggest a relative brachycephalisation, or broadening of the middle part of the vault, in Ukrainian populations coincident with the transition to an agricultural lifestyle. Brachycephalisation has been a recurrent observation in the physical anthropology literature, both as a continuous process throughout human evolution^48^,^49^ and as an abrupt shift at the Neolithic transition^10^,^50^. Conceptualised at the beginning of 20^th^ century, in a context typifying crania through indices based on a few linear dimensions, brachycephalisation, in the strict sense, is the change in proportion of maximum cranial breadth and maximum cranial length. However when considered in the context of the entire cranium, its definition may be expanded to describe a general broadening of the cranial vault, as observed in the Ukrainian data through a strong positive loadings of maximum cranial breadth (M8) and biauricular breadth (M11), and negative loadings of base length (M5), suggesting broadening of both the superior (traditionally-defined brachycephalisation) and inferior parts of the vault. It should be noted that this is effectively the opposite response that was noted in Nubian populations, where crania were observed to become proportionally longer and taller across the Neolithic transition^9^.

A number of hypotheses have been proposed to explain brachycephalisation. Diet has often been implicated in cranial shape determination for two distinct reasons. First, harder foods require stronger mastication, which in turn applies greater stress on the cranial skeleton leading to a more pronounced development of regions directly or indirectly associated with mastication. This may also have repercussions throughout the cranial structure as has been demonstrated in a number of organisms^51^,^52^,^53^. In human-specific studies, diet-associated brachycephalisation has been observed in a hunter-gatherer versus agriculturalist context^6^,^9^, and has traditionally been interpreted mechanically. However, diet has also been suggested to influence cranial morphology (as well as other anatomical characteristics) through the response of skeletal development to modified nutrient intake^8^,^54^,^55^,^56^,^57^,^58^. Menéndez *et al*.^57^ suggested that nutrient composition in the diet had a stronger impact on cranial diversification than did mechanical toughness. In contrast, recent work by Noback^8^ found diet to be of great importance to cranial morphology as a whole. However, this is not a simple dichotomy of farmer versus hunter-gatherer, as considered here and in many similar studies, but rather, it is a function of relative inputs of animal and plant foods ingested, suggesting that a number of intricate processes may be at work in parallel^8^. As such, the shift from a hunter-gatherer to an agricultural diet is likely to alter both the mechanical and physiological properties of diet.

Considering a very different set of processes, Billy^59^,^60^ and Schwidetzky^61^ found that higher levels of endogamy coincided with brachycephalisation and general skeletal gracilisation. Although these results have been criticised^62^, the transition to an agricultural lifestyle is likely to have resulted in more endogamous populations, as recently demonstrated through genetic studies^63^. However, whether changes in cranial morphology are a direct consequence, or the result of responses to a range of other environmental changes associated with changes in mating patterns, remains debated. Regardless, as the transition to an agricultural lifestyle has resulted in deep demographic changes^64^, if these were reflected in cranial morphology, that may be one of the factors driving the observed change in the Ukrainian vaults.

Despite all these potential factors that may have led to brachycephalisation in the Ukraine, the data for the Levant and Iberia show no signs of this process, although previous studies have suggested a potential brachycephalisation across the Neolithic transition for these two regions^10^,^47^. Unlike these two earlier studies, the methods employed here explicitly incorporate errors associated with sample size and missing data, possibly indicating that previous results may reflect sampling or preservation biases.

Nevertheless, the lack of change in cranial morphology observed here may be the result of a continuity of environmental conditions, leading to a relative constancy in behaviours, for example a lack of change in masticatory regime resulting from a diet whose consistency remained broadly similar across the transition, as has been reported for the Levant based on microwear patterns^30^,^31^. Alternatively, as the cranial vault has been demonstrated to reflect large-scale neutral genetic drift^23^,^65^,^66^, similar morphologies observed before and after the transition in the Levant and Iberia may reflect long-term genetic continuity in these regions. Although there is archaeological evidence for this being the case in the Levant^67^, the Neolithic transition in Iberia is unlikely to have taken place without the influx of any foreign populations, as molecular studies suggest a genetic discontinuity between Mesolithic and Neolithic populations in Iberia^68^,^69^. In addition, an influx of foreign populations coinciding with the Ukrainian groups considered as agricultural is not to be excluded as a potential causation of morphological change. Indeed, there is a mounting body of genetic evidence suggesting a mixed European Mesolithic and Caucasus origin for the Yamnaya culture^70^,^71^. Altogether, it is likely that the onset of agriculture in each of the three regions can be characterised by a unique set of features, with similarly idiosyncratic morphological responses in the cranium.

Error bars on the estimates cover a large proportion of the total observed range of morphological variation all plots (supplementary Figs S1–6) due to comparisons across datasets with widely varying sampling intensities (as is inevitably the case when comparing pre and post-agricultural transition groups). Consequently, the analysis of temporally and/or geographically more dense datasets may be able to reveal subtler patterns that are undetectable in the present data set. It is possible that the suggestion by Roseman^72^ is applicable here: that, although there should be informative cranial variability present, it is generally drowned out by large degrees of background noise.

## Conclusion

The transition from a hunter-gatherer to an agricultural lifestyle has resulted in the modification of many aspects of the human life. Most obviously, this includes changes in sources of primary subsistence products, but it can also encompass important changes in demography, population genetics, and physical environment, all of which can have profound impacts on skeletal morphology. However, the results for three western Eurasian regions presented here suggest a lack of consistent morphological change across the Neolithic transition in either the face or cranial vault. The variable characteristics of the three transition events considered suggest that morphological variability potentially associated solely with the fundamental cultural features associated with subsistence is obscured by that caused by other factors impacting cranial shape. Conditions specific to the Ukrainian transition enabled the detection of a general brachycephalisation, and may have dietary, demographic or genetic causes. The previous detection of similar patterns in other regions^10^,^47^,^50^ suggests that this may indeed be a universal change, but one which can remain undetected in many situations as a result of the multiple determinants of cranial morphology.

## Materials and Methods

The data were derived from the database previously used by Pinhasi and von Cramon-Taubadel^18^,^73^, augmented for pre-transition groups with data from Brewster *et al*.^74^. Three independent regions were selected, such that they were geographically distant from each other, and therefore likely to be free of significant amounts of shared gene-flow. These comprise 1) the Levant and Anatolia (“Levant”), where agriculture developed *in situ*^67^; 2) Iberia, which is the most south-western region in the trajectory of the spread of agriculture into Europe and in which agricultural communities first appear ca. 6,000 cal. BC^32^; and 3) the Ukraine, a region in which pottery appears as part of a hunter-gatherer subsistence spectrum and the first communities who adopt management of domesticated plants and animals appear ca. 4,500 cal. BC^75^, but in which full food-producing farming economies appear a millennium later^26^. The number of individuals analysed for each region is detailed in Table 3.

A set of 14 standard craniometric measurements was chosen to assess overall variations in cranial vault and face morphology, each represented by 7 variables (described in detail in Table 4). For both elements, measurements of height, breadth and length were included. Furthermore, the choice of variables chosen was influenced by their availability in the original database, minimising the instances of missing data. This is reflected in the choice of Basion-Prosthion length (M40) as a measure of facial length. Although it is a measure that also encompasses many elements of basal length, it was the only variable available for a large number of individuals. Furthermore, variable M11 (biauricular breadth) was replaced with variable M11b for the Levant dataset. This reflects a difference in measurement methodology of inferior vault breadth, but it enables the use of a larger dataset. All variables were size-adjusted though dividing them by the individual-specific geometric mean of the cranial element.

We considered two main archaeological categories: 1) pre-agricultural populations (“pre-transition”), which are represented by cultures that exhibit no evidence of a Neolithic lifestyle (such as the “Epipaleolithic” in the Levant and the “Mesolithic” in Iberia and the Ukraine); and 2) Neolithic agriculturalists (“post-transition”) (Table 3). These include populations that have adopted a Neolithic subsistence spectrum involving extensive reliance on the consumption of domesticated crops and animals. As the transition took place in very different ways in the three regions considered here, determination of pre- and post-transition categories required the consideration of local cultural circumstances. In the case of cultural groups with ambiguous characteristics having only adopted the agricultural lifestyle partially, these were omitted from analyses. In Iberia, the local discrete character of the transition resulted in no transitional group, and as such no individuals were excluded. In the Levant dataset, Pre Pottery Neolithic individuals were excluded, as they had adopted the subsistence element, but not many of the important technological aspects of the transition to agriculture, such as pottery. Finally, the individuals belonging to the Dnieper-Donets culture were excluded from the Ukrainian dataset, as, despite adoption of many material elements of the agricultural lifestyle, their subsistence remained mostly based on wild resources.

As is frequently the case when working with archaeological material, many of the crania were incomplete, requiring selection of the morphological variables to maximise the number of crania preserving these features, and therefore minimise the need for estimating missing data. Each anatomical partition (face and vault) was considered separately in the creation of two datasets for each geographic region. In each case, specimens missing more than 50% of the variables for a specific partition were discarded. The remaining missing data were estimated based on weighted averages of linear regressions. Linear regressions of all possible combinations of variables within the dataset were performed, providing a set of potential formulae for the estimation of missing data points from those elements that were preserved on an individual specimen^76^. Log-likelihoods for each formula were obtained using the standard deviations of the residuals. From the equation sets used to estimate a missing datum based on preserved features, proportional likelihoods were used as weightings in averaging the regression estimates for all applicable formulae for preserved features^76^. The R function for performing missing data estimation has been archived at CRAN (https://cran.r-project.org/) as the “WaverR” package.

Statistical analyses were carried out separately for each of the three regions and each partition. Principal component analyses (PCAs) were carried out on the pre-transition groups, creating a base morphospace for each dataset. Subsequently, post-transition groups were projected on their respective pre-transition morphospace using the loadings derived for each anatomical variable for each principle component (PC). Using this projected data, it was possible to identify potential presence of morphological shifts specific to a geographical region for either the facial or vault data partitions. A detailed look at the variable loadings enabled the morphological characterisation of the putative change.

Pairwise group comparisons were carried out for each of the three geographically defined datasets. However, there are large disparities in the pre- and post-transition sample sizes, indicating differential sampling and/or preservation across the Neolithic transition in all three geographic regions, which can bias inferences drawn from such data sets^77^,^78^. To account for potential biases due to sample size, we performed two resampling procedures on the data sets. First, the missing data estimation involved weighted regressions that themselves incorporate a certain degree of error. We resampled each estimate 10,000 times randomly assigning error to the estimate based on the standard deviation of the regression residuals. Second, we subsampled the individuals from the larger of the two samples down to the size of the smaller 10,000 times. These procedures allowed for an estimate of the amount of uncertainty around our reconstructions of pre- and post-transition cranial morphologies.

The significance of potential differences in distribution of the pre and post-transition groups was assessed by comparing the 2.5 percentile, median and 97.5 percentile of the two categories for each element in each region. This comparison was carried out by assessing the position of the pre-transitional value on histograms of the resampled post-transitional data.

## Additional Information

**How to cite this article**: Cheronet, O. *et al*. Morphological change in cranial shape following the transition to agriculture across western Eurasia. *Sci. Rep.* **6**, 33316; doi: 10.1038/srep33316 (2016).

## Supplementary Material

Supplementary Information

Supplementary Information

## Figures and Tables

**Table 1:** Loadings on the first two principal components of the PCAs of the pre-transition vaults.

Variable	PC1			PC2		
	Ukraine	Iberia	Levant	Ukraine	Iberia	Levant
M1	**−0.4821**	**−0.5801**	**0.8527**	0.3296	**0.7372**	0.2560
M5	**−0.5808**	**−0.3857**	**0.3113**	0.0509	**−0.4325**	**−0.4029**
M8	**0.3956**	**0.4616**	−0.1905	−0.0975	0.1598	−0.3267
M9	0.2446	0.0375	−0.2449	**0.5811**	0.0547	**0.5791**
M11	**0.3822**	**0.4581**	−0.2314	−0.1985	0.0969	0.3451
M12	0.0943	0.1516	−0.0915	−0.0998	0.1381	−0.0623
M17	−0.2426	−0.2596	−0.1338	**−0.7016**	**−0.4609**	**−0.4551**
% variance	54.54	41.42	40.63	17.25	21.10	21.73

Highlighted are the loadings above an absolute value of 0.35. Note that for the Levant dataset variable M11b was used in place of variable M11.

**Table 2:** Loadings on the first two principal components of the PCAs of the pre-transition faces.

Variable	PC1			PC2		
	Ukraine	Iberia	Levant	Ukraine	Iberia	Levant
M40	0.2405	−0.2056	−0.0132	**0.9172**	**0.4584**	−0.0079
M45	**0.8906**	**−0.9054**	−0.0357	−0.3300	−0.3483	−0.0050
M48	−0.2481	0.1207	0.0745	−0.1092	**−0.6341**	0.0026
M51	0.0836	−0.1905		−0.0192	0.2828	−0.1411
M52	−0.1588	0.1016	0.2597	−0.1622	0.0874	**−0.7249**
M54	0.0445	0.0698	**−0.5066**	0.0660	0.1246	**0.4523**
M55	−0.2308	0.2681	**0.7295**	−0.0826	**−0.4041**	**0.5000**
% variance	49.22	49.06	56.69	35.64	25.69	24.50

Highlighted are the loadings above an absolute value of 0.35.

**Table 3:** Number of specimens used in the analyses, including the pre and post-transition proportion for each group in brackets.

	Face (pre/post)	Vault (pre/post)
Ukraine	432 (34/280)	472 (39/309)
Iberia	102 (22/80)	120 (30/90)
Levant	157 (23/100)	174 (23/117)

**Table 4:** Description of variables used.

Martin Number^79^	Name	Skull Element	Definition
M1	Maximum cranial length	Vault	Maximum glabello-occipital length
M5	Skull base length	Vault	Basion to nasion
M8	Maximum cranial breadth	Vault	Widest measurement perpendicular to the medio-sagittal plane
M9	Least frontal breadth	Vault	Distance between left and right frontotemporale
M11	Biauricular breadth	Vault	Distance between left and right auriculare
M11b	Biradicular breadth	Vault	Distance between left and right radiculare
M12	Biasterionic breadth	Vault	Distance between left and right asterion
M17	Basi-bregmatic height	Vault	Distance between basion and bregma
M40	Basion-Prosthion length	Face	Basion to prosthion
M45	Bizygomatic breadth	Face	Distance between left and right zygion
M48	Upper facial height	Face	Nasion to prosthion
M51	Orbital breadth	Face	Maxillofrontale to ektokonchion
M52	Orbital height	Face	Greatest height of orbit, perpendicular to M51
M54	Nasal breadth	Face	Maximal nasal breadth
M55	Nasal height	Face	Nasion to Nasospinale
