# Supplementary material for: Morphological change in cranial shape following the transition to agriculture across western Eurasia
Source: Sci Rep. 2016 Sep 13;6:33316. doi: 10.1038/srep33316 (PMC5020731; doi:10.1038/srep33316)
Supplement: Supplementary Information [file srep33316-s1.pdf]

Morphological change in cranial shape following the transition to agriculture across western Eurasia

Olivia Cheronet[a,b*], John A. Finarelli[b,c], Ron Pihnasi[a,b]

a: School of Archaeology, University College Dublin, Belfield, Dublin 4, Ireland

b: Earth Institute, University College Dublin, Belfield, Dublin 4, Ireland

c: School of Biology and Environmental Sciences, University College Dublin, Belfield, Dublin 4, Ireland

Supplementary figures

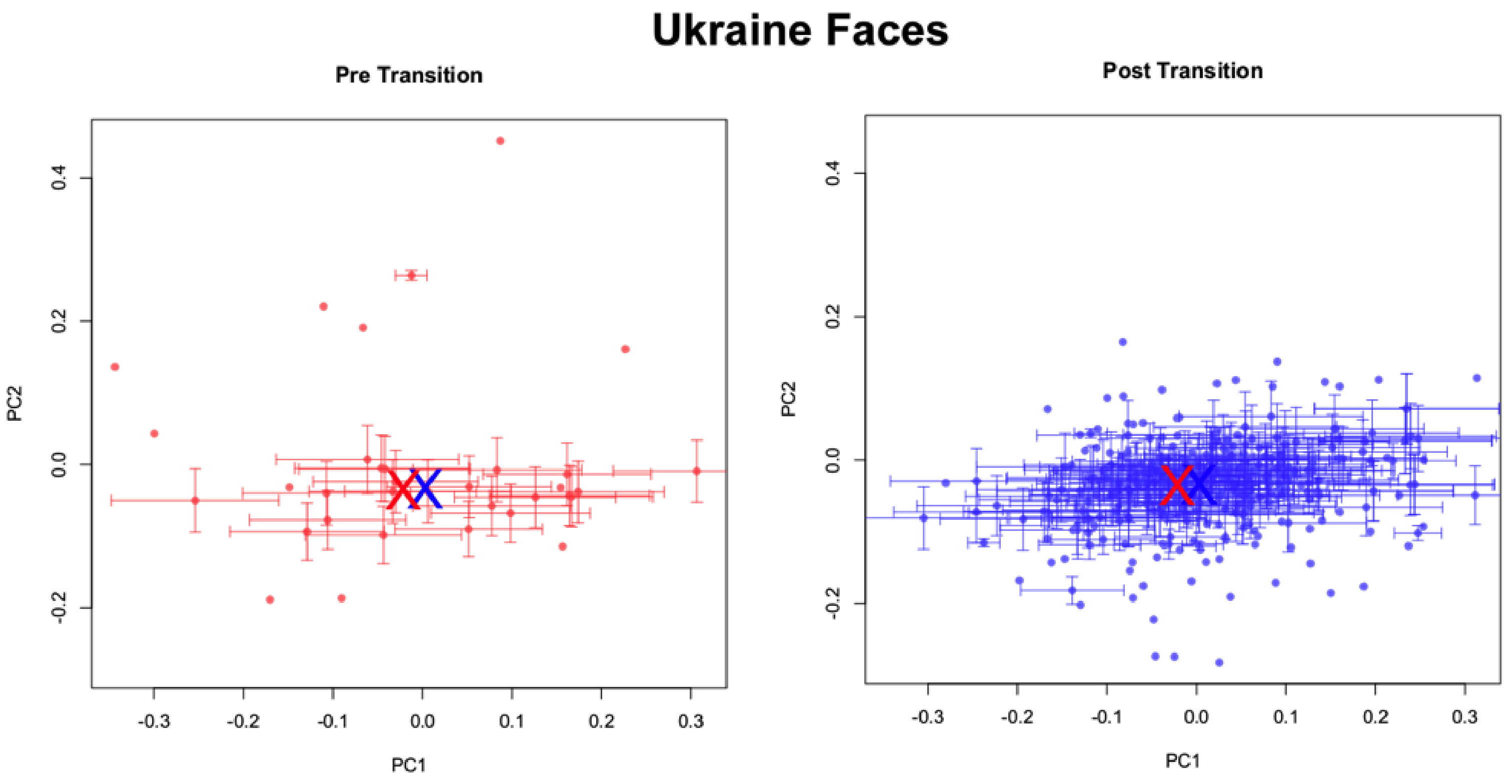

Supplementary figure S1: Bivariate plots of the first two principal components projection of the Ukrainian post-transition faces on a PCA of the Ukrainian pre-transition faces. Blue: distribution of the post-transition group. Red: distribution of the pre-transition group. For both the crosses are the centroids. The errors associated to the pre-transition individuals are shown on the left and with the post-transitional groups on the right.

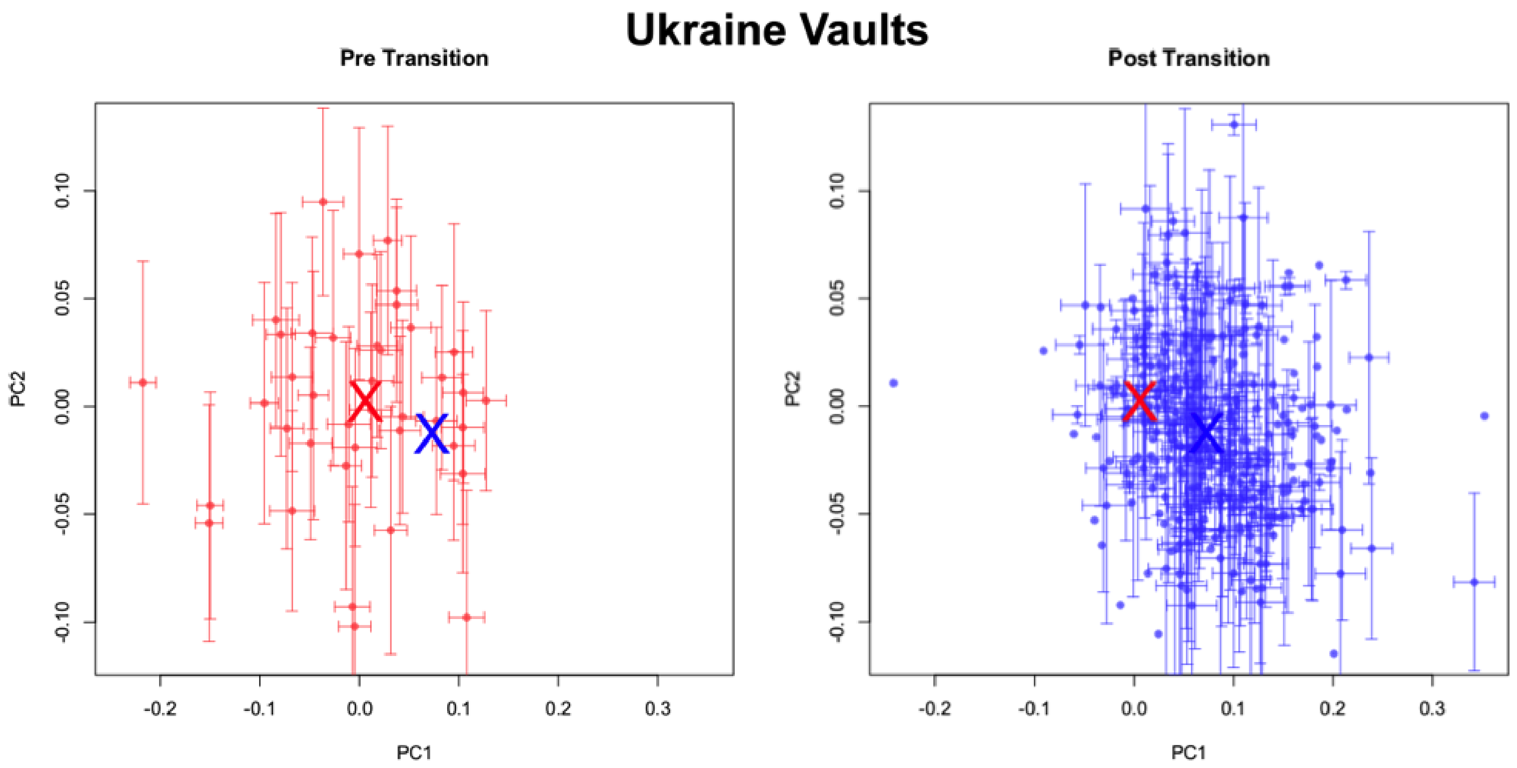

Supplementary figure S2: Bivariate plots of the first two principal components projection of the Ukrainian post-transition vaults on a PCA of the Ukrainian pre-transition faces. Blue: distribution of the post-transition group. Red: distribution of the pre-transition group. For both the crosses are the centroids. The errors associated to the pre-transition individuals are shown on the left and with the post-transitional groups on the right.

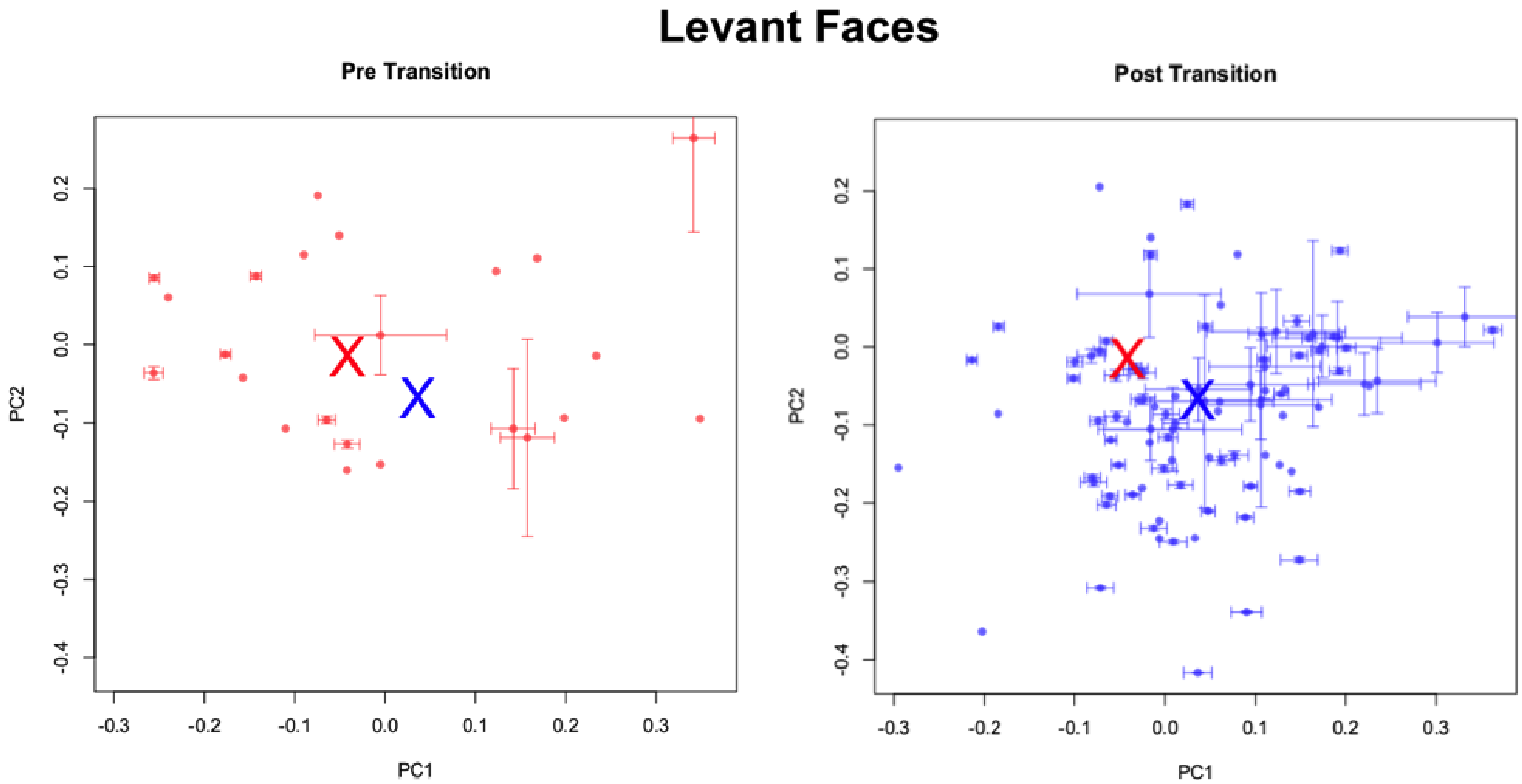

Supplementary figure S3: Bivariate plots of the first two principal components projection of the Levant post-transition faces on a PCA of the Levant pre-transition faces. Blue: distribution of the post-transition group. Red: distribution of the pre-transition group. For both the crosses are the centroids. The errors associated to the pre-transition individuals are shown on the left and with the post-transitional groups on the right.

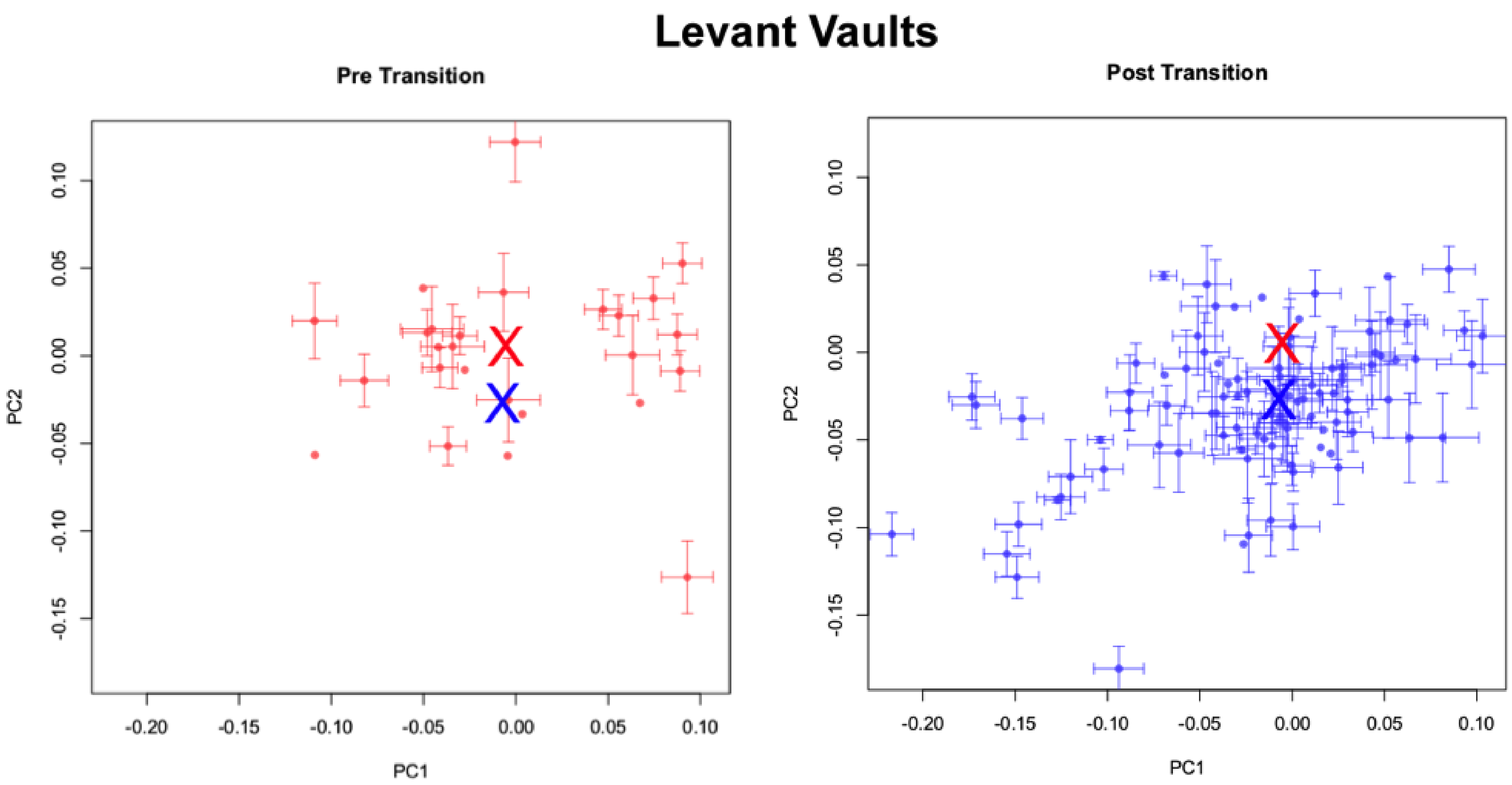

Supplementary figure S4: Bivariate plots of the first two principal components projection of the Levant post-transition vaults on a PCA of the Levant pre-transition vaults. Blue: distribution of the post-transition group. Red: distribution of the pre-transition group. For both the crosses are the centroids. The errors associated to the pre-transition individuals are shown on the left and with the post-transitional groups on the right.

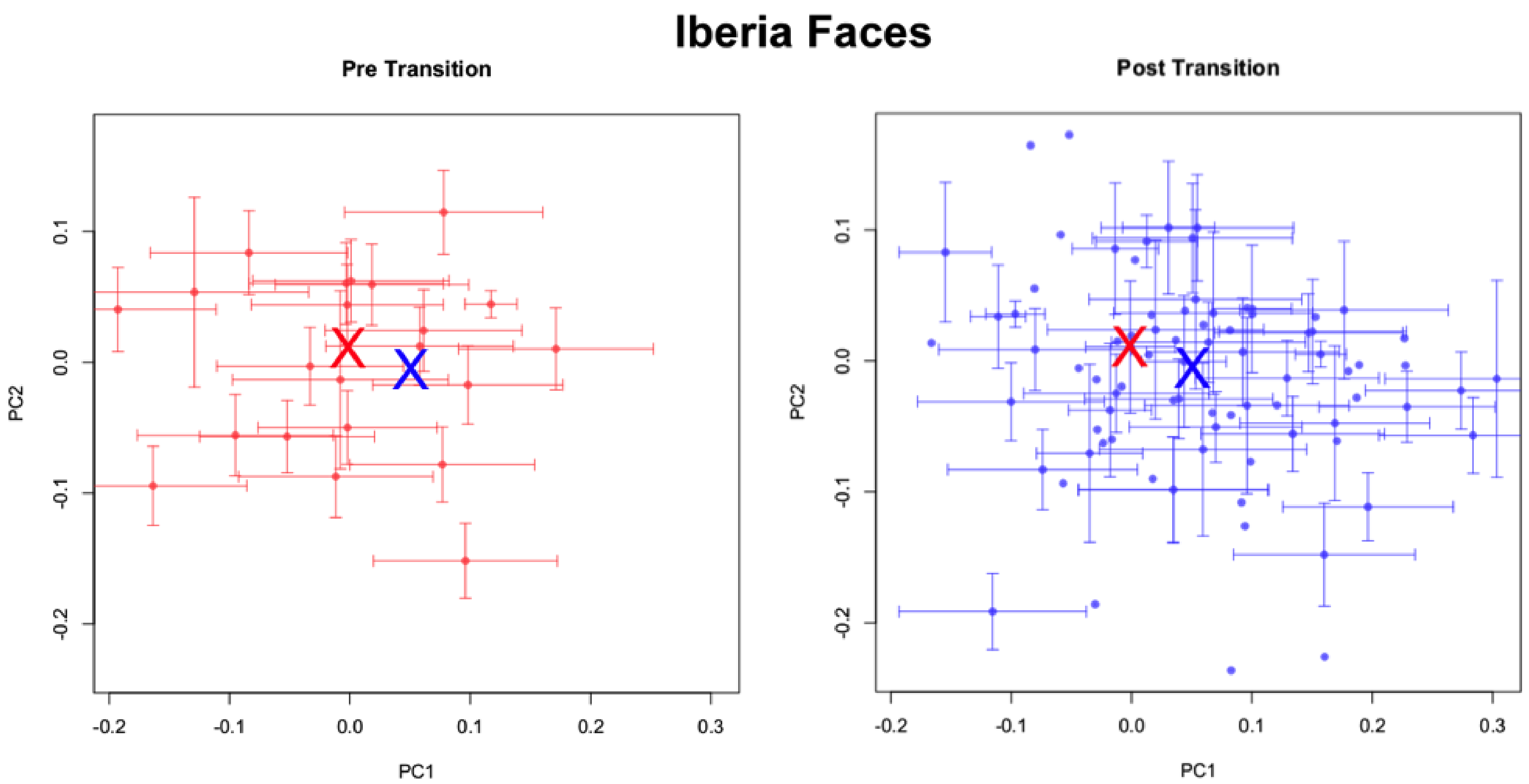

Supplementary figure S5: Bivariate plots of the first two principal components projection of the Iberia post-transition faces on a PCA of the Iberian pre-transition faces. Blue: distribution of the post-transition group. Red: distribution of the pre-transition group. For both the crosses are the centroids. The errors associated to the pre-transition individuals are shown on the left and with the post-transitional groups on the right.

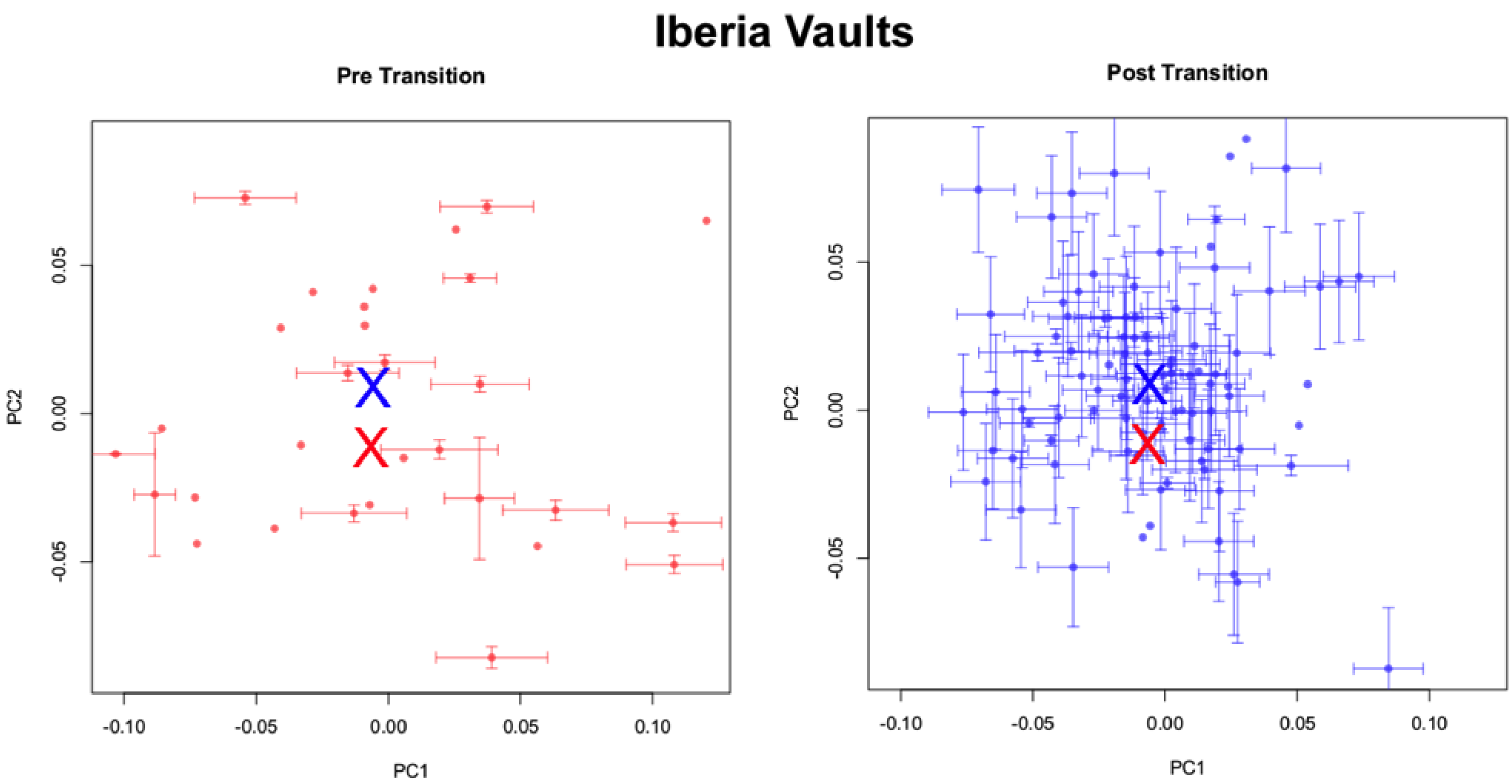

Supplementary figure S6: Bivariate plots of the first two principal components projection of the Iberian post-transition vaults on a PCA of the Iberian pre-transition vaults. Blue: distribution of the post-transition group. Red: distribution of the pre-transition group. For both the crosses are the centroids. The errors associated to the pre-transition individuals are shown on the left and with the post-transitional groups on the right.

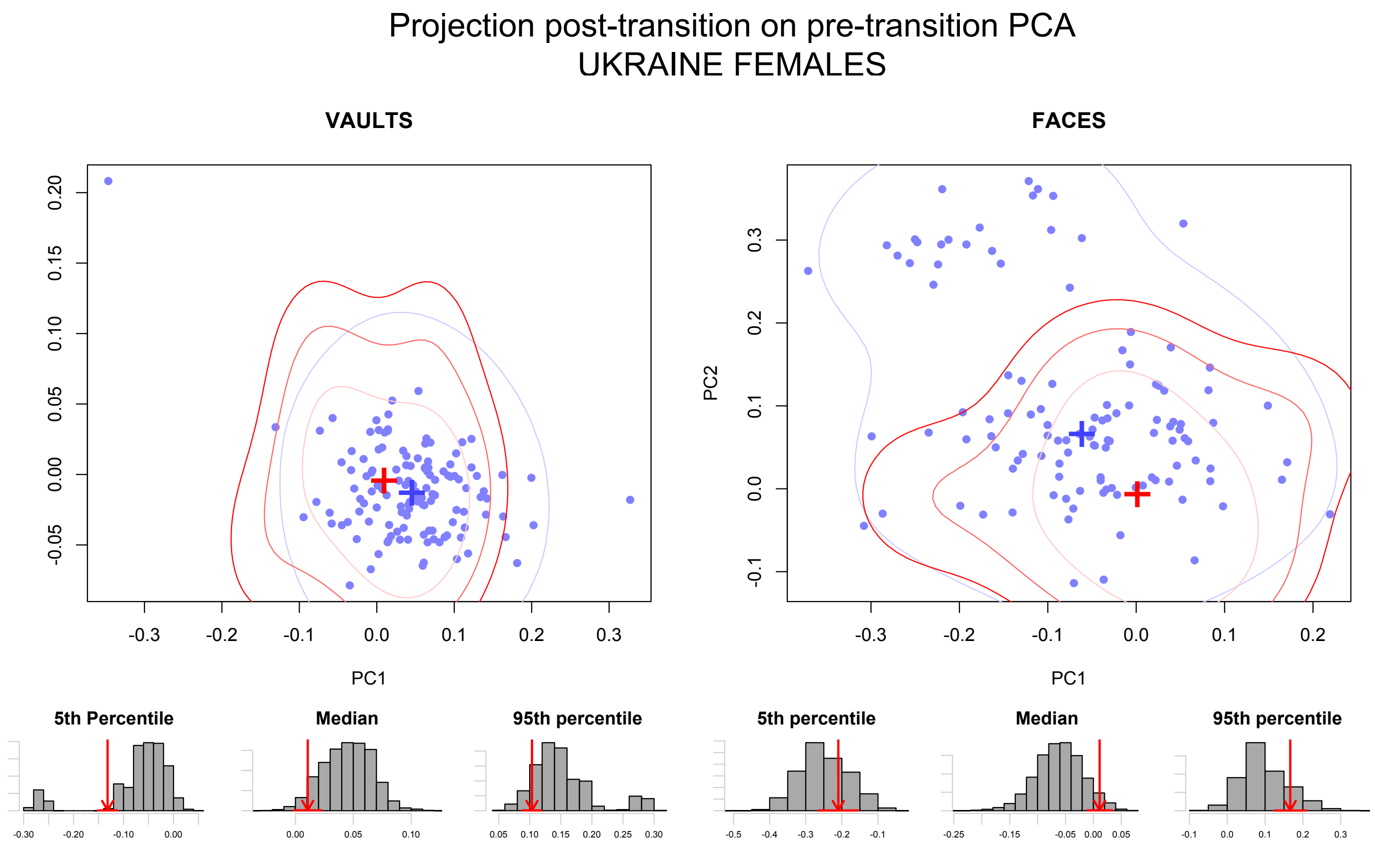

Supplementary figure S7: Analysis of Ukrainian females only. Bivariate plots of the first two principal components projection of the post-transition group on a PCA of the pre-transition group (A) Vaults, B) Faces). Blue: distribution of the post-transition. Red: distribution of the pre-transition group. For both groups, the crosses represent the mean shape and contours the distribution of 75% of the data. The histograms below illustrate the differences in pre and post transition groups distributions. For the 2.5 percentile, the mean and the 97.5 percentile, the bars represent the distribution of the larger post-transition resampled to the size of the pre-transition group, and the red arrow the value of the pre-transition group (for which the error associated with the data estimation process is expressed by the red horizontal line).

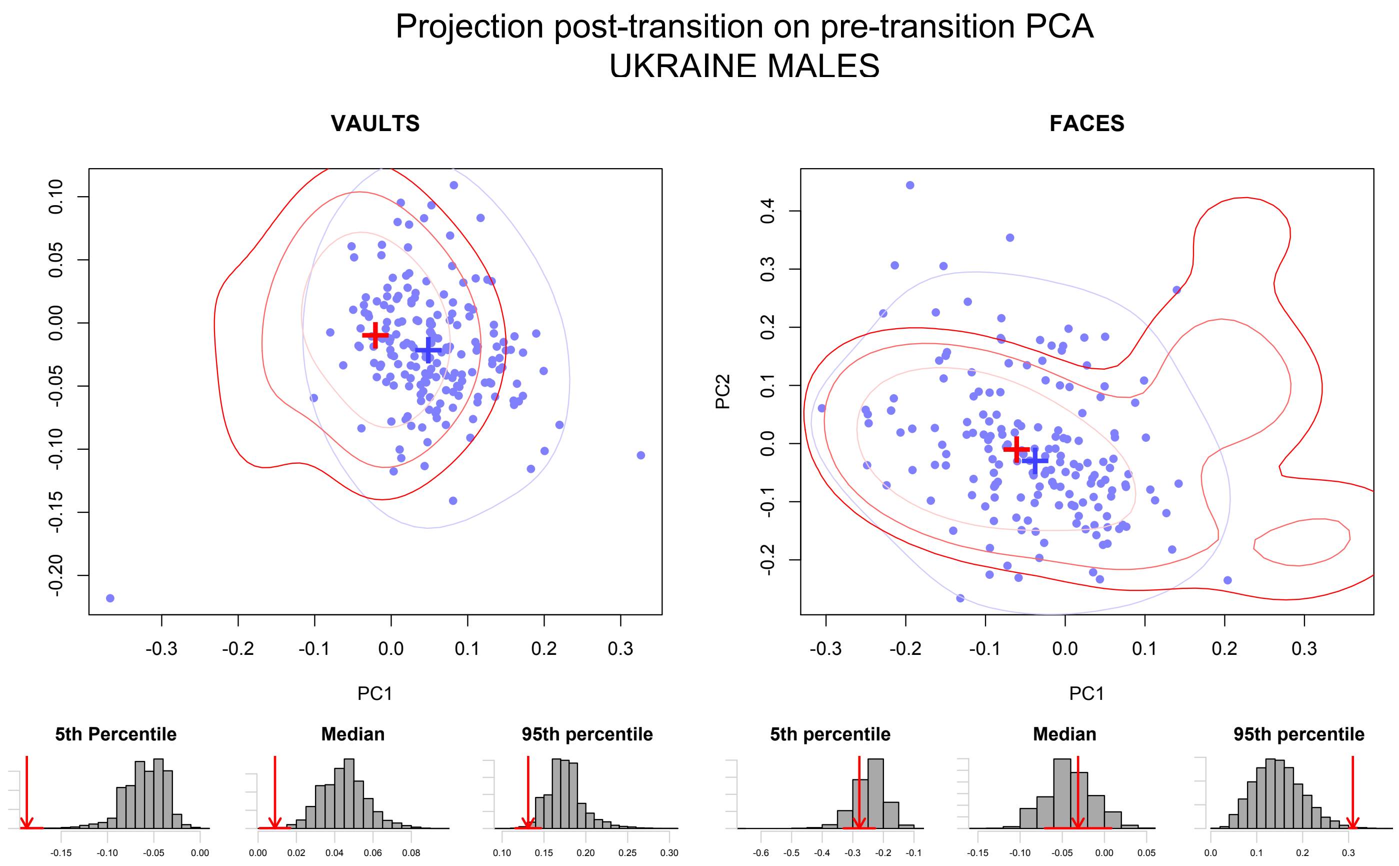

Supplementary figure S8: Analysis of Ukrainian males only. Bivariate plots of the first two principal components projection of the post-transition group on a PCA of the pre-transition group (A) Vaults, B) Faces). Blue: distribution of the post-transition. Red: distribution of the pre-transition group. For both groups, the crosses represent the mean shape and contours the distribution of 75% of the data. The histograms below illustrate the differences in pre and post transition groups distributions. For the 2.5 percentile, the mean and the 97.5 percentile, the bars represent the distribution of the larger post-transition resampled to the size of the pre-transition group, and the red arrow the value of the pre-transition group (for which the error associated with the data estimation process is expressed by the red horizontal line).
